# Supplementary material for: Attentional and executive functions in children and adolescents with developmental coordination disorder and the influence of comorbid disorders: A systematic review of the literature
Source: PLoS One. 2021 Jun 4;16(6):e0252043. doi: 10.1371/journal.pone.0252043 (PMC8177544; doi:10.1371/journal.pone.0252043)
Supplement: S1 Data — (DOCX) [file pone.0252043.s001.docx]

**Minimal data set**

# Method

This systematic review was performed based on the guidance outlined in the Preferred Reporting Items for Systematic Reviews and Meta-Analysis (PRISMA)^[1]^.

## Search Strategy and Selection Criteria

A systematic literature research was performed in PubMed/Medline and PsycINFO databases, including articles published between January 1980 and May 2020. Considering that pediatrics neuropsychology was poorly developed before 1980, it was chosen as the earliest publication date. Due to the multiple ways of conceptualizing attentional and executive functions in terms of their components, and to be as inclusive as possible, our research was made using all terms previously mentioned that are used to describe these functions. Thereby, research was conducted in English with the following keywords: (1) “developmental coordination disorder” OR “dyspraxi*” OR “motor skills disorder” OR “specific developmental disorder of motor function” OR “clumsiness” OR “clumsy child syndrome”; (2) AND “executive function*” OR “goal setting” OR “set-shifting” OR “shifting” OR “switching” OR “flexibility” OR “planning” OR “inhibit*” OR “working memory” OR “organis*” OR “organiz*” OR “self-regulation” OR “fluency” OR “attention*”; (3) AND “child*” OR “adolescen*” OR “teen*” OR “youth” OR “schoolchild*” OR “preschool*”. Publications referenced in the included articles were also screened to find additional articles.

Studies were included if (1) their participants were children or adolescents (17 years of age or younger; studies including older participants were excluded), (2) they had a group of participants with a diagnosis of DCD made by a health care professional, by a score at or below the 5^th^ percentile on the *Movement Assessment Battery for Children* (MABC first or second edition), as this score indicates a significant movement difficulty, or by DSM (IV, IV-TR or 5) criteria for DCD combined with a movement ability measure, in which case a total score at or below the 15^th^ percentile on the MABC was accepted, (3) their participants did not explicitly have any medical condition that could affect their motor or cognitive abilities, (4) they measured one or more attentional or executive functions using performance tests, (5) they used normative data of standardized measures or a control group comprising healthy individuals for results comparison, (6) they were published in French or English in a peer-reviewed journal and (7) they had an empirical research design.

Studies were first selected according to their title. Second, abstracts were read by two authors (CL and MPL) and studies that did not meet the eligibility criteria listed above were excluded. Then the same two authors screened full articles independently to ensure that all eligibility criteria were met. Their disagreements were discussed to reach a consensus and, whenever necessary, another author (SL) settled. The remaining articles were entirely read by the first author.

## Data Extraction

Data extraction from articles that met the selection criteria was made by the two first authors. Information was organized in an spreadsheet and included: title of the article, authors, year of publication, journal in which it was published, aims of the study, groups, their origin and samples size, gender and age of the subjects assessed (mean, standard deviation and range, when available), country in which the study took place, inclusion and exclusion criteria, presence of comorbid disorders in the sample and their nature, information about assessment of motor functions and confounding variables, cognitive functions assessed and tasks used, statistical analysis, results, limitations and commentaries about the paper. Subsequently, the relevant information was analyzed and summarized in Table 1: the authors and year of publication, the sample size, the gender of the participants and the number of males, the mean age of the participants, the presence of comorbid disorders, the attentional or executive function studied, the task(s) used to evaluate the function, and a summary of the results. Components of attentional and/or executive functioning assessed in each included study were determined according to what the study purported to measure. When the same score on a task was reported to assess more than one component of attentional or executive functioning, or to measure different components in different studies, results were reported only in relation to the component it was the most associated with, according to the *Compendium of Neuropsychological Tests*^[2]^ or the task’s reference. When several scores were available on the same task and associated with different components of attentional or executive functioning, information provided by each score was considered as a measure of its respective component.

## Quality Assessment of Included Studies

Quality assessment of studies included in this review was conducted using a checklist we developed based on the Newcastle-Ottawa Quality Assessment Scale (NOS)^[3]^. The NOS consists of three domains: “selection of subjects”, “comparability of subjects” and “outcome”, each domain including two to three items. Although the use of the NOS has been established in meta-analyses^[4]^, it was first developed for case-control and cohort studies. Therefore, its use was not suitable for the present study. Since we could not find a tool giving standardized criteria for assessing the quality of neuropsychological and behavioral studies, we adapted the NOS based on the methods used by Wu et al.^[5-7]^ and Caçola et al.^[8]^, that were inspired by the NOS and the PRISMA standards. We developed three to four quality items for each domain of the NOS (selection, comparability and outcome). The result is a 10-item checklist, including: inclusion/exclusion criteria and samples source (for the selection domain), comparability of samples regarding age, gender and IQ (for the comparability domain), and description of outcome measures, adequacy of outcome analysis and discussion (for the outcome domain; see Appendix 1 for the complete checklist). Items could be answered by “yes” or “no”, and quality level of evidence was rated as high (8 “yes” or more), medium (6-7 “yes”) or low (5 “yes” or less). The quality assessment was carried out by the first two authors independently and any discrepancy was discussed until they reached a decision by consensus.

**References**

1. Moher, D., Liberati, A., Tetzlaff, J., Altman, D. G., & Prisma Group. (2009). Preferred reporting items for systematic reviews and meta-analyses: the PRISMA statement. *PLoS Med*, *6*(7), e1000097. doi:10.1371/journal.pmed.1000097

2. Strauss, E., Sherman, E. M., & Spreen, O. (2006). *A compendium of neuropsychological tests: Administration, norms, and commentary* (3^rd^ ed.). New York, NY: Oxford University Press

3. Wells, G. A., Shea, B., O’Connell, D., Peterson, J., Welch, V., Losos, M., & Tugwell, P. (2014). Newcastle-Ottawa quality assessment scale; Cohort studies. Available from http://www.ohri.ca/programs/clinical_epidemiology/oxford.asp (accessed April 15, 2019)

4. Stang, A. (2010). Critical evaluation of the Newcastle-Ottawa scale for the assessment of the quality of nonrandomized studies in meta-analyses. *Eur J Epidemiol, 25*(9), 603-605. doi:10.1007/s10654-010-9491-z

5. Wu, M., Brockmeyer, T., Hartmann, M., Skunde, M., Herzog, W., & Friederich, H. C. (2016). Reward-related decision making in eating and weight disorders: A systematic review and meta-analysis of the evidence from neuropsychological studies. *Neurosci Biobehav Rev*, *61*, 177-196. doi: 10.1016/j.neubiorev.2015.11.017

6. Wu, M., Hartmann, M., Skunde, M., Herzog, W., & Friederich, H. C. (2013). Inhibitory control in bulimic-type eating disorders: a systematic review and meta-analysis. *PloS One*, *8*(12), e83412. doi:10.1371/journal.pone.0083412

7. Wu, M., Brockmeyer, T., Hartmann, M., Skunde, M., Herzog, W., & Friederich, H. C. (2014). Set-shifting ability across the spectrum of eating disorders and in overweight and obesity: a systematic review and meta-analysis. *Psychol Med*, *44*(16), 3365-3385. doi: 10.1017/S0033291714000294

8. Caçola, P., Miller, H. L., & Williamson, P. O. (2017). Behavioral comparisons in autism spectrum disorder and developmental coordination disorder: a systematic literature review. *Res Autism Spectr Disord*, 38, 6-18. doi:10.1016/j.rasd.2017.03.004
