# Supplementary material for: Attentional and executive functions in children and adolescents with developmental coordination disorder and the influence of comorbid disorders: A systematic review of the literature
Source: PLoS One. 2021 Jun 4;16(6):e0252043. doi: 10.1371/journal.pone.0252043 (PMC8177544; doi:10.1371/journal.pone.0252043)
Supplement: S1 Appendix — (DOCX) [file pone.0252043.s003.docx]

# S1 Appendix

Quality assessment:

Adaptation of the Newcastle-Ottawa Quality Assessment Scale

Cohort studies

**Selection**

1. The study mentions how diagnoses in all clinical groups were made and inclusion criteria for each group.
2. The study mentions exclusion criteria for each group.
3. The study mentions the sample sources of every group.
4. All groups are drawn from the same community.

**Comparability**

1. All groups are age-matched.
2. All groups are gender-matched.
3. All groups are IQ-matched or the study controls for IQ.

**Outcome**

1. The study mentions neuropsychological tests and/or clearly describes experimental tasks used to assess attentional/executive functioning, and references about them are provided.
2. Outcome analysis is adequate (statistical measures, conclusions according to results).
3. The study presents a clear discussion considering implications and limitations of outcomes.

Answer items by “yes” or “no”.

8 “yes” or more = high quality level of evidence

6-7 “yes” = medium quality level of evidence

5 “yes” or less = low quality level of evidence
