# Supplementary material for: Attentional and executive functions in children and adolescents with developmental coordination disorder and the influence of comorbid disorders: A systematic review of the literature
Source: PLoS One. 2021 Jun 4;16(6):e0252043. doi: 10.1371/journal.pone.0252043 (PMC8177544; doi:10.1371/journal.pone.0252043)
Supplement: S2 Appendix — (DOCX) [file pone.0252043.s004.docx]

# S2 Appendix

Summary of attentional and executive functions assessed, age ranges, sample sizes and tasks used among studies

|  | Attentional or executive functions studied | Ages of DCD groups in which function was assessed (years)  Range (mean) | Total DCD sample size in which function was assessed | Tasks used to assess attentional or executive function | Studies  Authors (years) |
| --- | --- | --- | --- | --- | --- |
| Attentional functions | Alertness and sustained attention | 7-16 (10.5) | 157 |  |  |
|  | - Visual | 7-16 (10.7) | 131 | CPT-II (omission errors) | Biotteau et al. (2017) |
|  |  |  |  |  | Blais et al. (2017) |
|  |  |  |  | CPT double version (correct responses) | de Castelnau et al. (2007) |
|  |  |  |  | KITAP: Alerting | Kaiser & Albaret (2016) |
|  |  |  |  | Go/No Go task (omission errors) | Querne et al. (2008) |
|  |  |  |  | Go/No Go task with positively and negatively valenced stimuli (omission errors) | Rahimi-Golkhandan et al. (2016) |
|  |  |  |  | Visuospatial Working Memory Paradigm (non-delay condition) | Tsai et al. (2012) |
|  | - Auditive | 7-12 (8.8) | 26 | TEA-Ch: Score! | Williams et al. (2013) |
|  | Selective attention | 5-12 (8.0) | 160 |  |  |
|  | - Visual | 5-12 (7.5) | 93 | CAS: Expressive attention, Number Detection, Receptive Attention | Asonitou & Koutsouki (2016) |
|  |  |  |  |  | Asonitou et al. (2012) |
|  |  |  |  | NEPSY: Visual attention | Barray et al. (2008) |
|  |  |  |  | KITAP: Distractibility | Kaiser & Albaret (2016) |
|  | - Auditive | 5-12 (9.6) | 45 | NEPSY: Auditory attention and Response set | Barray et al. (2008) |
|  |  |  |  |  | Toussaint-Thorin et al. (2013) |
|  | Divided attention | 8-12 (9.9) | 7 | KITAP: Divided attention | Kaiser & Albaret (2016) |
| Executive functions | Inhibitory control | 3-17 (9.56) | 700 |  |  |
|  | Response inhibition | 3-17 (9.58) | 449 |  |  |
|  | - Nonverbal | 3-17 (10.8) | 431 | Motor VIMI (total errors) | Bernardi et al. (2017) |
|  |  |  |  |  | Leonard et al. (2015) |
|  |  |  |  | Motor VIMI (total errors and completion time) | Bernardi et al. (2016) |
|  |  |  |  | CPT-II (commission errors) | Biotteau et al. (2017) |
|  |  |  |  |  | Blais et al. (2017 |
|  |  |  |  | CPT double version (commission errors) | de Castelnau et al. (2007) |
|  |  |  |  | Go/No Go task (commission errors) | Dyck & Piek (2010)  Thornton et al. (2018) |
|  |  |  |  |  | Querne et al. (2008) |
|  |  |  |  | Go/No Go task with informative and uninformative precue conditions (initiation and failure-to-inhibit errors) | Mandich et al. (2003) |
|  |  |  |  | Go/No Go task with positively and negatively valenced stimuli (commission errors) | Rahimi-Golkhandan et al. (2016) |
|  |  |  |  | Go/No Go app (motor response; commission errors) | Sartori et al. (2020) |
|  |  |  |  | Visual Simon task (failure-to-inhibit errors) | Mandich et al. (2002) |
|  |  |  |  | NEPSY: Knock-Tap task | Pratt et al. (2014) |
|  |  |  |  | Double-Jump Reaching task – Modified version | Ruddock et al. (2016) |
|  |  |  |  |  | Ruddock et al. (2015) |
|  |  |  |  | The Paired Images test | Toussaint-Thorin et al. (2013) |
|  | - Verbal | 6-14 (7.9) | 147 | Verbal VIMI (total errors) | Bernardi et al. (2017) |
|  |  |  |  |  | Leonard et al. (2015) |
|  |  |  |  | Verbal VIMI (total errors and completion time) | Bernardi et al. (2016) |
|  |  |  |  | Go/No Go app (verbal response; commission errors) | Sartori et al. (2020) |
|  |  |  |  | Hayling Test Parts A & B | Sartori et al. (2020) |
|  |  |  |  | Stroop task | Alesi et al. (2019)  Pratt et al. (2014) |
|  | Attentional inhibition | 7-12 (9.7) | 271 |  |  |
|  | - Endogenous mode | 7-12 (9.8) | 188 | COVAT | Chen et al. (2012) |
|  |  |  |  |  | ^2^Tsai et al. (2009) |
|  |  |  |  |  | Wilson & Maruff (1999) |
|  |  |  |  |  | Wilson et al. (1997) |
|  |  |  |  | Endogenous Posner paradigm | ^1^Tsai et al. (2009) |
|  |  |  |  | Go/No Go task with both informative and uninformative precue conditions (reaction times) | Mandich et al. (2003) |
|  | - Exogenous mode | 7-12 (9.8) | 123 | COVAT | Wilson & Maruff (1999) |
|  |  |  |  |  | Wilson et al. (1997) |
|  |  |  |  | Visuospatial Attention task cued and non-cued conditions | Gonzalez et al. (2016) |
|  |  |  |  | Visuospatial Attention (eye-gaze cueing) paradigm | Tsai et al. (2010) |
|  |  |  |  |  | Wang et al. (2015) |
|  |  |  |  | Visual Simon task (reaction times) | Mandich et al. (2002) |
|  |  |  |  |  | Tsai, Yu et al. (2009) |
|  | Working memory | 3-14 (7.6) | 378 |  |  |
|  | - Visuospatial | 5-14 (7.8) | 243 | AWMA: Odd-One-Out, Mr. X, Spatial Span | Alloway (2007) |
|  |  |  |  |  | Alloway (2011) |
|  |  |  |  |  | Alloway & Archibald (2008) |
|  |  |  |  |  | Alloway et al. (2009) |
|  |  |  |  |  | Alloway & Temple (2007) |
|  |  |  |  | Odd-One-Out test | Bernardi et al. (2017) |
|  |  |  |  |  | Leonard et al. (2015) |
|  |  |  |  |  | Sartori et al. (2020) |
|  |  |  |  | Visuospatial Working Memory Tasks | Alesi et al. (2019) |
|  |  |  |  | Visuospatial Working Memory Paradigm (delay condition) | Tsai et al. (2012) |
|  | - Verbal | 3-14 (7.6) | 354 | AWMA: Listening Recall, Counting Recall, Backwards Digit Recall | Alloway (2007) |
|  |  |  |  |  | Alloway (2011) |
|  |  |  |  |  | Alloway & Archibald (2008) |
|  |  |  |  |  | Alloway et al. (2009) |
|  |  |  |  |  | Alloway & Temple (2007) |
|  |  |  |  | Oral Word Span in Sentences | Sartori et al. (2020) |
|  |  |  |  | Verbal Working Memory Tasks | Alesi et al. (2019) |
|  |  |  |  | WMTBC: Listening Recall | Bernardi et al. (2017) |
|  |  |  |  |  | Leonard et al. (2015) |
|  |  |  |  | WISC-IV: Digit Span, Letter-number Sequencing | Biotteau et al. (2017) |
|  |  |  |  |  | Sumner et al. (2016) |
|  |  |  |  | Trailmaking/Memory Updating task, Goal Neglect task | Dyck & Piek (2010) |
|  |  |  |  |  | Piek et al. (2007) |
|  | Planning | 5-14 (9.0) | 230 |  |  |
|  | - Nonverbal | 7-14 (11.0) | 40 | D-KEFS: Sorting test (nonverbal sorts) | Bernardi et al. (2017) |
|  |  |  |  |  | Leonard et al. (2015) |
|  | - Verbal | 7-14 (11.0) | 40 | D-KEFS: Sorting test (verbal sorts) | Bernardi et al. (2017) |
|  |  |  |  |  | Leonard et al. (2015) |
|  | - General | 5-14 (8.4) | 190 | CAS: Matching Numbers, Planned Codes, Planned Connections | Asonitou & Koutsouki (2016) |
|  |  |  |  |  | Asonitou et al. (2012) |
|  |  |  |  | NEPSY: Tower task | Barray et al. (2008) |
|  |  |  |  |  | Pratt et al. (2014) |
|  |  |  |  |  | Toussaint-Thorin et al. (2013) |
|  |  |  |  | River Crossing task | Kirby et al. (2010) |
|  |  |  |  | Rotational Bar task | Pratt et al. (2014) |
|  |  |  |  | BADS-C: 6-Part test | Toussaint-Thorin et al. (2013) |
|  | Cognitive flexibility | 6-14 (9.5) | 117 |  |  |
|  | - Nonverbal | 7-14 (11.0) | 23 | CANTAB: Intra-/Extra-Dimensional Shift | Bernardi et al. (2017) |
|  |  |  |  |  | Leonard et al. (2015) |
|  | - Verbal | 7-14 (11.0) | 23 | D-KEFS: Trail Making test | Bernardi et al. (2017) |
|  |  |  |  |  | Leonard et al. (2015) |
|  | - General | 6-14 (9.1) | 94 | Visual Inspection Time task | Piek et al. (2007) |
|  |  |  |  | Five Digits Test | Sartori et al. (2020) |
|  |  |  |  | Trail Making test A and B for Children | Sartori et al. (2020) |
|  |  |  |  |  | Toussaint-Thorin et al. (2013) |
|  | Fluency | 5-14 (7.5) | 86 |  |  |
|  | - Nonverbal | 5-14 (10.4) | 55 | NEPSY: Design Fluency | Barray et al. (2008) |
|  |  |  |  | D-KEFS: Design Fluency | Bernardi et al. (2017) |
|  |  |  |  |  | Leonard et al. (2015) |
|  | - Verbal | 7-14 (7.7) | 54 | D-KEFS:Verbal Fluency | Bernardi et al. (2017) |
|  |  |  |  |  | Leonard et al. (2015) |
|  |  |  |  | BVN-5-11 Verbal Fluency | Alesi et al. (2019) |
|  |  |  |  | NEPSY: Verbal Fluency | Toussaint-Thorin et al. (2013) |
|  | General executive functioning | 6-12 (9.1) | 52 | Ecological assessment: cooking task | Toussaint-Thorin et al. (2013) |
|  |  |  |  | Wisconsin Card Sorting Test | Zhu et al. (2012) |

Note: CPT-II = Continuous Performance Test, Second Edition; KITAP = computerized test battery of attention for children; TEA-Ch = Test of Everyday Attention for Children; CAS = Das-Naglieri Cognitive Assessment System; NEPSY = Developmental Neuropsychological Assessment; VIMI = Verbal Inhibition Motor Inhibition test; COVAT = Covert Orienting of Visuospatial Attention Task; AWMA = Automated Working Memory Assessment; WMTBC = Working Memory Test Battery for Children; WISC-IV = Wechsler Intelligence Scale for Children, Fourth Edition; D-KEFS = Delis-Kaplan Executive Function System; BADS-C = Behavioural Assessment of the Dysexecutive Syndrome in Children; CANTAB = Cambridge Neuropsychological Test Automated Battery; BVN-5-11 = Development Neurological Assessment Battery.
