# Supplementary material for: Attentional and executive functions in children and adolescents with developmental coordination disorder and the influence of comorbid disorders: A systematic review of the literature
Source: PLoS One. 2021 Jun 4;16(6):e0252043. doi: 10.1371/journal.pone.0252043 (PMC8177544; doi:10.1371/journal.pone.0252043)
Supplement: Attachment — Submitted filename: Response_reviewers_Round1.docx [file pone.0252043.s005.docx]

Reviewer #1: Title: Attentional and Executive Functions in Children and Adolescents with Developmental Coordination Disorder and the Influence of Comorbid Disorders: A Systematic Review of the Literature. The research justification and aim are clear and relevant; the article is good and useful to improve the future intervention. The article seems solid and the language helps keep track.

Q1. No important or relevant references appear in the abstract, it has to improve.

R1. Thank you for your commentary. However, according to the PLOSONE guidelines, no reference should be included in the abstract.

Q2. It must be verified that the set of Citations used in the text are correctly cited in the References section (and vice versa) and that, in each case, the DOI or URL corresponding to each cited work is located. The references inserted in the text must also be verified according to APA regulations.

R2. Some DOIs were indeed missing, and the ones that could be found were added in the references. The authors also reviewed and corrected the in-text citations according to APA regulations, and we hope that everything is in order.

Q3. INTRODUCTION. The theoretical review that begins in the introduction of the article reflects a deep exploration, but it would be interesting to use recent studies (last 5 years).
Perhaps, expressions such as "most recent" should be reviewed if there are other subsequent references later.

R3. Thank you for the suggestion. It is true that older studies are used to define dyspraxia and DCD in the introduction. The authors decided on older references because they reflect the theoretical models of DCD that have been adopted and accepted since they were first developed. However, the term “recent” was indeed not appropriate for studies published more than 5 years ago. Accordingly, the sentence was changed to (Pg. 5):

“The one proposed by Vaivre-Douret and her team (2011) offers a thorough definition as it stipulates that dyspraxia involves a deficit of both execution of voluntary gesture and planning/programming of movement.”

Q4. Regarding what is already known about the subject of the article, it is recommended to specify why it is an important study and how it differs from previous publications.

R4. The rationale behind the study was briefly mentioned on Pg. 9, but it is true that it needed more emphasis. Accordingly, the sentence was changed to (Pg. 9):

“This review is an important step in the acquisition and gathering of supplemental evidence regarding the attentional and executive deficits found in DCD. Indeed, consolidating the results found in previous research appears relevant, especially given that several studies based their conclusions on small samples.”

Q5. The research question should be added clearly in the introduction. Also, the objective that appears in the summary does not match (or is misleading) with what appears as a research question in the objectives. It would be interesting to improve this aspect.

R5. Thank you for these observations. Regarding the research question, the section in which is it stated was renamed “**1.4 State of knowledge**” (Pg. 9) in order to make it clear that it is indeed in the introduction.

As for the objective in the summary, it was changed to (Pg.2):

“Previous studies have reported deficits in visuospatial/nonverbal and planning tasks. This systematic review of the literature aims to determine if impairments can be found in other attentional and executive functions as well.”

Q6. Methods. The methodology is appropriate in terms of the selection criteria. The variables involved are clearly defined. They should review the description of the content in table 1, since what appears in table 1 partially coincides with what is discussed in the methodology section.

R6. Thank you for the suggestion. A sentence was added in the “Methodology” section to describe the content included in Table 1 (Pg. 12):

“Subsequently, the relevant information was analyzed and summarized in Table 1: the authors and year of publication, the sample size, the gender of the participants and the number of males, the mean age of the participants, the presence of comorbid disorders, the attentional or executive function studied, the task(s) used to evaluate the function, and a summary of the results.”

Q7. There are enough details to replicate the study but it would be interesting to add the two or three elements that the NOS includes in each domain and why it was not suitable for the present study.

R7. The reason why the NOS could not be used in the present study was added to the text (Pg. 13):

“Although the use of the NOS has been established in meta-analyses (Stang, 2010), it was developed for case-control and cohort studies. Therefore, its use was not suitable for the present study.”

Since the quality items for the present study are mentioned and described, the authors decided not to add the NOS items in the text. The NOS reference is provided, and readers can access it if need be.

Q8. Results. The results are arranged by categories, which makes it easier to read and understand. The text is not repetitive with respect to the data in the table. Statistically significant results are clear in some categories, it is recommended to do this in all categories.

R8. The provided p values of the statistically significant results have been added in Table 1.

Q9. In the sustained attention category, the relationship between DCD and ADHD is sometimes confusing. It would be convenient to clarify this.

R9. In accordance with the comment of another reviewer, the “Results” section has been modified in an extensive way. As such, the relationship between DCD and ADHD has been explained more clearly.

Q10. Although the data is presented clearly and the table format is correct, the table is presented in alphabetical order according to authors, the results are arranged according to categories.

R10. We agree that it might confuse the readers. This is why Appendix 2 had been added to the article. It shows the results presented according to categories. We hope that, together, Appendix 2 and Table 1 offer a clear presentation of the results.

Q11.The quality items should be specified in table 2 to facilitate reading.

R.11. Thank you for pointing that out. The quality items were added to the table.

Q12. Discussion and conclusions. It is recommended to relate the content of the discussion with what was previously stated in the introduction and avoid explaining future prospects in this section.

R12. The authors agree with that statement. Accordingly, the “Future research” section was moved after the “Discussion” section.

Q13. Future research adds that future studies should also focus on children under 8 and over 12 years old, why?

R13. This is a good question. As mentioned previously in the “Discussion”, not many studies have focused on children under 8 and over 12 years old. It would be interesting to have more data for those age groups.

Reviewer #2: The present work shows the results obtained from a systematic review of the literature whose main objective is to identify the attentional and executive deficits present in children with developmental coordination disorder (DCD), for which the type of cognitive tasks, the modality of the tasks (verbal and non-verbal) and the influence of comorbid disorders on the attentional and executive profiles were systematically considered. The presented results contribute to a better understanding of the cognitive profile associated with CDD, revealing as one of the main findings to be considered that executive impairments are common in CDD (especially in certain areas). Therefore, one of the main implications to be taken into account, and which emerges from the review conducted in this paper, is the consideration of possible attentional and executive impairments in CDD children as inherent to the disorder itself, which, in itself, is one of the strengths of this review. Another of the main strengths of this review is at the methodological level, as it is a comprehensive review that includes a wide variety of research terms and different types of experimental tasks, as well as standardized neuropsychological tests for the selection of studies. Another strength is the consideration of studies using samples of DCD children with comorbid disorders.

Q1. A possible limitation arising from the above consideration is that at certain moments and, assuming the importance of presenting the information in the exhaustive way in which it is done, the reading of the document becomes certainly cumbersome and complex. It would be desirable to review this issue.

R1. The authors agree that the article might be overwhelming for the readers, and considering that Appendix 2 and Table 1 provide exhaustive summaries of the results, we decided to improve the “Results” section to make it less complex.

Q2. Other possible limitations that may be found in this work are assumed by the authors themselves in the section described for this purpose, such as the creation of their own quality assessment tool or the inclusion of studies that, basically due to the number of samples, cannot be considered relative. In this case, the authors warn the reader to take the conclusions drawn with sufficient caution. Furthermore, in the section on Directions for Future Research, lines of research are suggested that also respond to another series of limitations that can be found in the review carried out, such as including studies of self-regulation, extending the age of the sample, verifying whether the executive deficit is prolonged or whether it is a developmental delay, or verifying the overlap between executive functions and motor skills. In this sense, it is desirable that the conclusions section appears before Strengths and Limitations and Directions for Future Research.

R2. Thank you for the suggestion. Accordingly, the “Strengths and limitations” and “Future research” sections were moved after the “Conclusions” section.
